# Psoriasis: Obesity and Fatty Acids

**DOI:** 10.3389/fimmu.2019.01807

**Published:** 2019-07-31

**Authors:** Manfred Kunz, Jan C. Simon, Anja Saalbach

**Affiliations:** Department of Dermatology, Venereology and Allergology, University of Leipzig, Leipzig, Germany

**Keywords:** psoriasis, obesity, fatty acids, metabolism, genetics

## Abstract

Psoriasis is chronic inflammatory skin disease affecting skin, joints, cardiovascular system, brain, and metabolism. The pathogenesis of psoriasis is mediated by a complex interplay between the immune system, inflammatory mediators of different pathways, e.g., TNF-alpha and the IL-23/IL-17 pathways, psoriasis-associated susceptibility loci, autoantigens, and multiple environmental factors. Psoriasis is triggered by the combination of genetic and environmental factors. A novel environmental risk factor with rising importance is obesity. Several studies proved that obesity is an independent risk factor for the onset and severity of psoriasis. Due to the dramatic increase of obesity worldwide this minireview focuses on obesity as a major environmental risk factor for psoriasis and the mechanisms of obesity-mediated exacerbation of psoriasis.

## Introduction

Psoriasis is a polygenic chronic inflammatory skin disease (1–3). A large proportion (20–30%) of the psoriasis patients suffer from additional joint involvement mainly affecting the distal extremities but also larger joints (3). Plaque-type psoriasis, the most common disease variant, which is seen in ~85% of cases, commonly manifests as dull-red, erythematous, scaly plaques particularly on the extensor surfaces of elbows, knees, and on the scalp. Less common psoriasis subtypes include pustular, guttate, inverse, erythrodermic, and palmoplantar psoriasis (4).

Psoriasis has a significant genetic background as shown by the enhanced risk for the development of the disease in offsprings and siblings of psoriasis patients and familial occurrence (5, 6). Genetic associations in psoriasis vulgaris were mainly described for the major histocompatibility complex (MHC) locus on chromosome 6 carrying the human leukocyte antigen (HLA) genes and other immune-regulatory genes such as complement factors and TNF-α (6). The strongest association was observed for the HLA-C allele Cw6, a classical HLA class I allele that was found in 46% of psoriasis patients but only in 7% of a control population (7). Subsequent genome-wide linkage studies by microsatellite analysis provided a further set of possible genomic regions with linkage to psoriasis such as the *PSORS1* locus and other, non-MHC loci such as *PSORS2-5* loci (8). More recently performed genome-wide association (GWAS) studies on psoriasis vulgaris have identified several additional psoriasis risk factors that comprise genes associated with chronic inflammation including *IL12B* (9, 10), *IL23A* and *IL23R* (9), *IL2/IL21* (7), *TNFAIP3* and *TNIP1* (9), *ZNF313* (11), and epidermal/antimicrobial genes such as *SLC12A8* and *HBD* (human β-defensin gene) (12) and the LCE (late cornified envelope) gene cluster (10).

In contrast to psoriasis vulgaris, pustular psoriasis shows genetic associations with mutations in the *IL36RN* gene with the strongest association for generalized psoriasis pustulosa and a weaker association for palmoplantar pustulosis and acrodermatitis continua of Hallopeau (13). Palmoplantar pustulosis shows higher prevalence in female subjects and smokers. Guttate psoriasis is associated with environmental factors such as stress and infections, but no distinct genetic background has been defined so far. The different pathogenic mechanisms may also impact on treatment response, e.g., guttate psoriasis is less responsive to treatment with anti-TNF antibodies than plaque-type psoriasis (14).

Psoriasis is currently regarded as an auto-immune disease because it shares many features with other autoimmune diseases such as chronicity of the clinical symptoms and chronic inflammation, involvement of TNF-α and a genetic background with overlapping gene loci with other auto-immune diseases (15, 16). Potential autoantigens such as keratin 17 with sequence homologies to streptococcal M-proteins, the antimicrobial peptide LL37 and the melanocytic autoantigen ADAMTSL5 have been identified recently. LL37 and ADAMTSL5 are recognized by T-cells after binding to HLA-C^*^06:02 underlining the role of distinct HLA genotypes in the pathogenesis of psoriasis (17–19).

The central pathogenic cell types in psoriasis are epidermal keratinocytes, antigen presenting cells, and inflammatory T cells with complex feedback mechanisms (1, 2, 20–22).

Dysregulation of this complex interplay of cells of the innate and adaptive immune system promotes the proliferation and attenuates the differentiation of epidermal keratinocytes resulting in the distinctive thickened, scaly plaques seen in psoriasis vulgaris. Psoriasis is mediated by a plethora of cytokines and chemokines where TNF-α and the IL-23/IL-17 axis play an outstanding role (20, 23). IL-23 which is produced by antigen-presenting cells supports the development of IL-17-secreting CD4+ memory T cells (Th17 cells). Differential expression of both components of IL-23 (IL-23p19 and IL-12p40) was observed in psoriatic skin lesions in contrast to non-involved skin. However, there were no significant differences for the IL-12p35 subunit, suggestive for a particular role of IL-23 and not IL-12 in psoriasis (24). Th17 cells, neutrophils and mast cells produce IL-17A which exerts a feedforward inflammatory response in keratinocytes by triggering chemokine and cytokine production in keratinocytes. IL17 also activates neutrophils, B cells, monocytes, and macrophages (25, 26). The feedforward keratinocyte responses are self-amplifying, resulting in sustained pathogenic immune infiltration and the development of mature psoriatic plaques. Interestingly, inflammatory bowel diseases, which show a significant association with psoriasis vulgaris, and where IL-17 cytokines play a major pathogenic role, do not respond to anti-IL-17 treatment, while psoriasis does. This may be due to the fact that IL-17A can also play a protective role in the intestinal tract under inflammatory conditions as shown in an experimental mouse model of colitis (25).

The role of IL-23 as a master regulator in psoriasis was highlighted by the induction of psoriasis-like ear swelling, epidermal hyperplasia and acanthosis upon injection of IL-23 into mouse ears, which was dependent on IL17- and IL-22 (27, 28). These findings were supportive for a role of IL-23, IL-17, and IL-22 in psoriasis. The role of these cytokines in psoriasis pathogenesis is further emphasized by the currently used highly effective treatment modalities for psoriasis and psoriasis arthritis using antibodies directed against TNF-α, IL-23p19, and IL-17 (4, 29, 30). The analysis of gene expression patterns in psoriasis lesional skin under treatment with biological agents showed that gene expression patterns of IL-23- and IL-17-induced genes were indeed reduced by treatment with an anti-IL-12/23 antibody in healing skin lesions (31).

The detrimental feedforward inflammatory process in psoriasis is not restricted to the skin. The uncontrolled inflammatory response contributes to a number of comorbid conditions in psoriasis including cardiometabolic disease, stroke, and metabolic syndrome (obesity, hypertension, dyslipidemia, and diabetes) (32–36).

In general, psoriasis is believed to be triggered by the combination of genetic and environmental factors. It has been accepted that the interplay between environmental and genetic factors contributes to the onset, development and clinical symptoms of psoriasis. A significant number of studies identified ultraviolet light, drugs, smoking, alcohol, and infections as well as mental and biomechanical stress as environmental risk factors affecting psoriasis by interfering with its genetic predisposition and immune response (37).

A novel risk factor for psoriasis of high socioeconomic importance is adiposity. Several studies have shown that obesity is an independent risk factor for the onset and severity of psoriasis (38, 39). Due to the dramatic increase of obesity worldwide, this minireview focuses on obesity as one environmental risk factors for psoriasis and the mechanisms of obesity-mediated exacerbation of psoriasis.

## Psoriasis and Obesity

The incidence of psoriasis among adults had almost doubled between the 1970s and 2000 (40). Since the genetic basis should have not significantly changed, environmental factors including the Western lifestyle might have played a role in this growing prevalence (41). The dietary habits in industrialized nations often support high-fat, high-salt, and high-sugar diets with excess caloric intake resulting in obesity and metabolic syndrome (42). In a current large population-based Norwegian study including close to 35,000 subjects, an association of metabolic syndrome with an increased risk to develop psoriasis has been described. The analysis of metabolic factors indicated that adiposity is a central factor in this association (43). Similar findings were reported by others [reviewed in (38, 39)]. It is difficult to show what comes first, psoriasis or obesity. Pronounced social isolation, poor eating habits, depression, increased alcohol consumption, and decreased physical activity in patients with psoriasis might explain how psoriasis might lead to obesity (38).

However, epidemiological studies provide strong evidence that obesity predisposes patients to psoriasis and amplifies psoriatic inflammation. A study of Setty and co-workers including 78,626 women (of whom 892 reported having psoriasis) indicated that adiposity and weight gain were risk factors for the development of psoriasis (44). Patients with a body mass index (BMI) of 35 or more had a relative increased risk for development of psoriasis of 2.69 compared to lean patients (44). A recent prospective study indicated that obesity and high abdominal fat mass doubled the risk of psoriasis (45). These studies suggest that preventing weight gain, promoting maintenance of a normal body weight, and reduction of body mass may reduce incidence of psoriasis. Indeed, several studies showed a positive impact of weight loss on the severity of psoriasis (46). Thus, dietary weight reduction with a hypocaloric diet is recommended in overweight and obese patients with psoriasis (47). An open question is whether differences in the type of diet (low carbohydrate, ketogenic, or vegan/vegetarian diets) have an effect on psoriasis improvement. Understanding the epidemiological relationship between obesity/nutrition and psoriasis is important to assess the relevance of the environmental factors as modifiable risk factors in psoriasis pathogenesis and to develop new strategies to support anti-psoriatic treatments (48).

Since adipose tissue is an important endocrine organ secreting soluble factors involved in inflammation and immunity, it has been postulated that adipose tissue expansion and its secretion of pro-inflammatory mediators might worsen psoriasis. High levels of resistin and leptin have been found in obese psoriasis patients (39). A recent meta-analysis showed that patients with psoriasis have higher levels of leptin compared to persons without psoriasis (49).

In addition, obesity alters the cellular composition and activity of inflammatory cells in the skin. Nakamizo and co-workers described an accumulation of IL-17A-producing γδ T cells in psoriatic skin lesions of high fat diet (HFD)-induced obese mice, which resulted in an exacerbation of psoriatic dermatitis (50). Moreover, genetically engineered diabetic (*db/db)* mice showed an enhanced psoriatic skin inflammation with enhanced levels of IL-17A and IL-22 (51). Another study showed that long-term HFD over 9 months promoted the accumulation of specific CD11c^+^ macrophages in the skin, in an epidermal fatty acid binding protein (E-FABP)-dependent manner (52). In elegant studies, Christ and co-workers showed that Western diet (WD) induces a long-lasting trained immunity in myeloid cells. The authors induced systemic inflammation in *Ldlr*^−/−^ mice by WD feeding that subsided after shifting mice to chow diet. WD induced long-lasting transcriptomic and epigenomic reprogramming of myeloid progenitor cells resulting in increased proliferation and innate immune responses (53).

Another important aspect is the fact that obesity and nutrition affect the microbiome (54, 55). Recently it has been shown that the microbiome -which stands for the entire microorganisms that live on human outer and inner body surfaces- exerts a strong influence on human autoimmune diseases (56). There is already some evidence that this might also be the case in psoriasis (57). The role of the microbiome for metabolic processes has also been emphasized in recent experimental studies (58). Interestingly, a correlation exists between the microbiome and IL-17 production in autoimmune diseases (59–61). Changes in the gut microbiome in psoriasis refer to a decrease in the *Bacteroidetes* phylum with an increase in the *Faecalibacterium* genus. It was suggested that bacteria shed their cell wall components, such as lipopolysaccharide and lipoteichoic acid into the blood stream thereby supporting a chronic inflammatory state. Along this line, pro-biotic substances have been shown to exert an influence on autoimmune diseases such as Crohn's disease, colitis ulcerosa, and rheumatoid arthritis but so far have not been tested for their impact on psoriasis (62).

## Psoriasis and Fatty Acids

**Figure 1:**
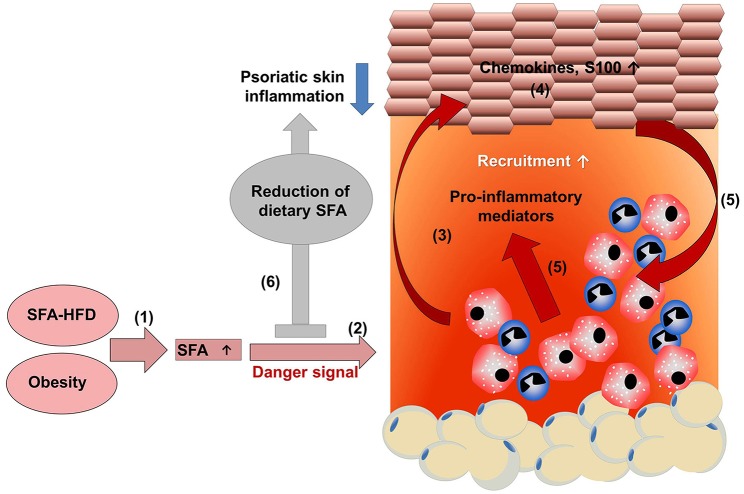
HFD-derived SFAs amplify psoriatic inflammation. **(1)** A diet rich in saturated fatty acids (SFAs) increases SFA serum concentration. **(2)** Chronic intake of a high fat diet increases adipose tissue, resulting in obesity with high SFA serum levels. **(2)** SFAs sensitize myeloid cells resulting in an amplified pro-inflammatory response with enhanced secretion of pro-inflammatory mediators in the presence of a danger signal. **(3)** The enhanced myeloid cell activation contributes to a disturbance of keratinocyte proliferation, differentiation, and **(4)** enhances the production of chemokines and S100 proteins. **(5)** Consequently, more myeloid immune cells are recruited into skin lesion and activated, further enhancing psoriatic skin inflammation. **(6)** Dietary reduction of SFAs dampens psoriatic skin inflammation, which might support treatment efficacy in psoriatic patients.

Interestingly, psoriatic patients on low energy diet showed a significant decrease of serum lipids in parallel to a reduction of skin involvement compared to a control group on a normal diet (63). However, body weight did not differ between both groups linking obesity and psoriasis independent of adipose tissue. In line with this, a recent study by our group using an imiquimod-induced psoriasis mouse model showed that specific dietary components, rather than obesity itself, may exacerbate psoriasis (64). In this study, a correlation of serum concentrations of free fatty acids (FFAs) with severity of psoriatic inflammation in HFD-induced obese mice was observed (64, 65). Interestingly, these data could be recapitulated in a human cohort of psoriasis patients where blood levels of FFAs correlated with the severity of psoriatic skin lesions (64). In accordance with these findings, in the above mentioned large population-based Norwegian study only blood fat levels and adiposity showed a positive association with psoriasis while glucose levels did not (43). To examine the causal relationship between FFAs and the HFD-induced exacerbation of psoriatic skin inflammation we fed mice for only 5 weeks (64). At this time point mice are lean and metabolically healthy while expressing elevated levels of serum FFAs. Psoriatic skin inflammation was strongly enhanced accompanied by an increased tissue infiltration of myeloid cells, epidermal thickening and expression of S100 proteins, chemokines, and pro-inflammatory cytokines such as IL-1β (64). Consistently, *ob/ob* mice, another model of murine obesity on normal diet, did not exhibit enhanced psoriatic skin inflammation (50). To further discriminate between the impact of adipose tissue and FFAs we used different dietary approaches (64). First, mice received standard HFD with high saturated FA (SFAs) for 2.5 weeks, which was then changed to a HFD with reduced SFAs but enriched polyunsaturated FAs (PUFA) content for additional 2.5 weeks. After 5 weeks, weight did not significantly differ between these groups but the modified HFD fed mice showed significantly lower SFA blood levels, and psoriatic inflammation was strongly reduced. Mice were then fed for 20 weeks with HFD to mimic the situation of obese patients. Then, the diet was switched to a low-fat chow diet without PUFA supplementation. Again, diet change decreased the serum concentration of SFAs without effect on weight. Importantly, these obese mice with reduced serum concentration of SFA exhibited a reduced psoriatic skin inflammation compared to obese mice on HFD (64). This study showed that dietary SFAs seem to be key amplifiers of psoriatic inflammation and suggest that restriction of SFAs may be beneficial for both lean and obese patients (Figure 1).

Due to limited evidence of a beneficial effect of fish oil for psoriasis, fish oil supplementation is not recommended for psoriasis treatment (47). Data from our study might explain the failure of PUFA supplementation as a therapeutic measure in psoriasis (66–69). It appears that a reduction of SFAs is more efficient than PUFA supplementation. At present, caloric restriction is recommended for overweight and obese psoriasis patients. Future clinical trials have to verify whether a specification of this recommendation—the reduction of SFAs as adjuvant dietary measure—might support conventional anti-inflammatory therapies.

## Fatty Acids and Inflammation

Long chain SFAs such as palmitate are enriched in states of nutrient excess and obesity (65, 70). SFAs can produce insulin resistance, endoplasmatic reticulum stress, oxidative stress, and cell death, a phenomenon referred to as lipotoxicity (70). They can bind to cell surface molecules such as CD36, free fatty acid receptors (FFAR1-4) and intracellular receptors/sensors [E-FABP and Peroxisome Proliferator Activated Receptor (PPAR) γ] that control inflammatory cell signaling and gene expression (71). SFAs are able to induce pro-inflammatory cytokines in human macrophages *via* pathways involving *de novo* ceramide synthesis (72). SFAs, but not unsaturated FAs, bind to E-FABP which activates retinoid acid receptor (RAR) resulting in differentiation of CD11c+ macrophages and expression of proinflammatory cytokines (73). It has been suggested that SFAs, via binding to TLR2 and TLR4, stimulate the expression of pro-inflammatory signaling pathways (74, 75). Current findings indicate that SFAs are not TLR4 agonists, but instead provide a second hit of activation that is dependent on prior TLR4 activation (76). Consistently, several studies did not detect any direct FFA-mediated activation of myeloid cells (64, 77, 78). However, amplification of the pro-inflammatory response of myeloid cells in the presence of SFAs has been described in many studies (64, 65, 72, 77–79). How can SFAs amplify the pro-inflammatory response?

PPARs are specialized receptors detecting FFA-derived signal molecules. Loss of PPAR-γ dampens *de novo* sterol biosynthesis and augments IFN-β production, which in turn suppresses the transcription of IL-1α and IL-1β in LPS-stimulated macrophages (80). Uptake of SFAs leads to enhanced ceramide generation, which in turn activates PKC-ζ and MAPK, resulting in increased IL-6 and IL-8 secretion upon LPS stimulation (79).

Elevated FFAs caused by HFD or obesity activate the NLRP3 inflammasome in macrophages resulting in increased IL-1β and IL-18 secretion (78). In the presence of danger signals SFAs induce inflammasome activation by induction of mitochondrial reactive oxygen species, or by stimulation of AMP-activated protein kinase, autophagy, or lysosome- and calcineurin-dependent pathways (70, 78). Excess SFAs uptake induces intracellular SFAs crystallization that leads to NLRP3 inflammasome activation and subsequent IL-1β release via lysosomal dysfunction (81).

**Figure 2:**
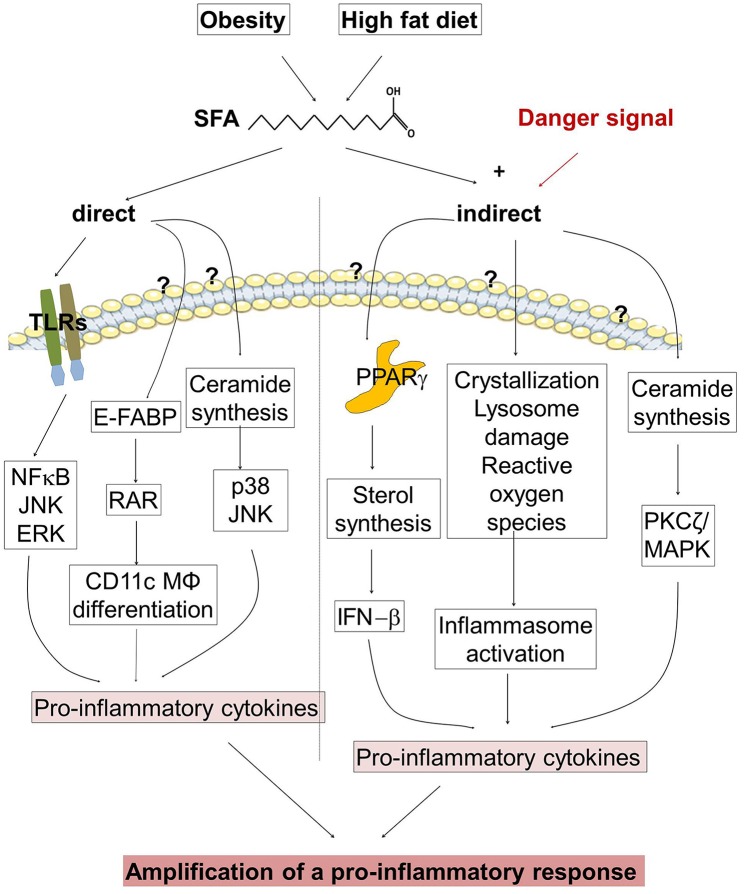
Regulation of the pro-inflammatory response in psoriasis. (**Left** half of the figure) Obesity and high fat diet (HFD) increase the concentration of saturated fatty acids (SFAs). SFAs are able to stimulate directly the expression of pro-inflammatory cytokines. SFAs activate toll-like receptors (TLR), and bind to cytoplasmic epidermal fatty acid binding proteins (E-FABPs) activating retinoid acid receptor (RAR) and stimulate the differentiation of CD11c+ macrophages (MØ). An Increase of SFAs modulates ceramide synthesis. (**Right** half of the figure) SFAs amplify the pro-inflammatory response in the presence of a danger signal. SFAs stimulate the expression of pro-inflammatory cytokines via binding to PPARs, by inflammasome activation, and by modulation of ceramide synthesis. Until now it is not clear which receptors are involved in FFA binding, translocation into the cell and subsequent pro-inflammatory activity.

Taken together, SFAs can amplify the pro-inflammatory response via direct and indirect actions (Figure 2). Thus, restriction of dietary SFAs might be helpful to suppress psoriatic inflammation.

## Conclusions

Psoriasis is a chronic inflammatory skin disease mediated by a complex interplay between immune cells and tissue resident cells. Genetic and environmental factors contribute to psoriasis pathogenesis. Environmental factors such obesity and nutrition have an important impact on onset and severity of psoriasis. Recent studies suggest that dietary SFAs seem to be key amplifiers of psoriatic inflammation and suggest that restriction of SFAs may be beneficial for both lean and obese patients. The clinical relevance has to be proven in future clinical trials to improve psoriasis treatment responses and co-morbidities.

## Author Contributions

AS and MK wrote the manuscript. JS edited and discussed the manuscript.

### Conflict of Interest Statement

MK received travel grants from UCB Pharma, and is member of advisory boards of Novartis Pharma and LEO Pharma. JS received speakers honoraria and is member of advisory boards of Novartis Pharma, Janssen Pharma, UCB Pharma, and AbbVie Pharma and received travel grants from these companies and from LEO Pharma. The remaining author declares that the research was conducted in the absence of any commercial or financial relationships that could be construed as a potential conflict of interest.
